# Cardiovascular inflammaging: Mechanisms, consequences, and therapeutic perspectives

**DOI:** 10.1016/j.xcrm.2025.102264

**Published:** 2025-08-08

**Authors:** Luke Spray, Gavin Richardson, Judith Haendeler, Joachim Altschmied, Valencia Rumampouw, Sienna B. Wallis, Georgios Georgiopoulos, Stephen White, Amanda Unsworth, Konstantinos Stellos, Simon Tual-Chalot, Ioakim Spyridopoulos

**Affiliations:** 1 Translational and Clinical Research Institute, Vascular Biology and Medicine Theme, Faculty of Medical Sciences, Newcastle University, Centre for Life, NE1 3BZ Newcastle Upon Tyne, UK; 2 Biosciences Institute, Vascular Biology and Medicine Theme, Faculty of Medical Sciences, Newcastle University, Centre for Life, NE1 3BZ Newcastle Upon Tyne, UK; 3 Cardiovascular Degeneration, Clinical Chemistry and Laboratory Diagnostics, Medical Faculty, University Hospital and Heinrich-Heine University Duesseldorf, 40225 Duesseldorf, Germany; 4 CARID, Cardiovascular Research Institute Düsseldorf, Medical Faculty, University Hospital and Heinrich-Heine University Duesseldorf, 40225 Duesseldorf, Germany; 5 Department of Physiology, School of Medicine, University of Patras, Patras, Greece; 6 Thrombosis Collective, Leeds Institute of Cardiovascular and Metabolic Medicine, Faculty of Medicine and Health, University of Leeds, Leeds, UK; 7 Department of Cardiovascular Research, European Center for Angioscience (ECAS), Medical Faculty Mannheim, Heidelberg University, Mannheim, Germany; 8 German Centre for Cardiovascular Research (DZHK), Partner Site Heidelberg/Mannheim, Mannheim, Germany; 9 Helmholtz-Institute for Translational AngioCardioScience (HI-TAC) of the Max Delbrück Center for Molecular Medicine in the Helmholtz Association (MDC) at the Heidelberg University, Heidelberg, Germany; 10 Department of Medicine, University Medical Centre Mannheim, Heidelberg University, Mannheim, Germany

**Keywords:** inflammation, inflammaging, cardiovascular disease, senescence, mitochondrial dysfunction

## Abstract

Both aging and systemic inflammation are major risk factors for cardiovascular disease. This review summarizes the interrelationship of aging and inflammation—known as inflammaging—and the consequences for cardiovascular health. We discuss mechanisms including epigenetic modification, mitochondrial dysfunction, cellular senescence, and gut dysbiosis, many of which are themselves interrelated. Increasing understanding of inflammaging provides an array of biomarkers, some of which are now recommended in international guidelines. We also discuss therapeutic strategies aiming to modify the process of inflammaging and improve cardiovascular disease outcomes, either with immunomodulating agents or with therapies targeted at specific mechanisms, such as senolytics, telomerase activators, and pre- and probiotic supplementation. We conclude that inflammaging is a key part of cardiovascular aging and provides encouraging opportunities for new therapies.

## Graphical abstract

**Figure:**
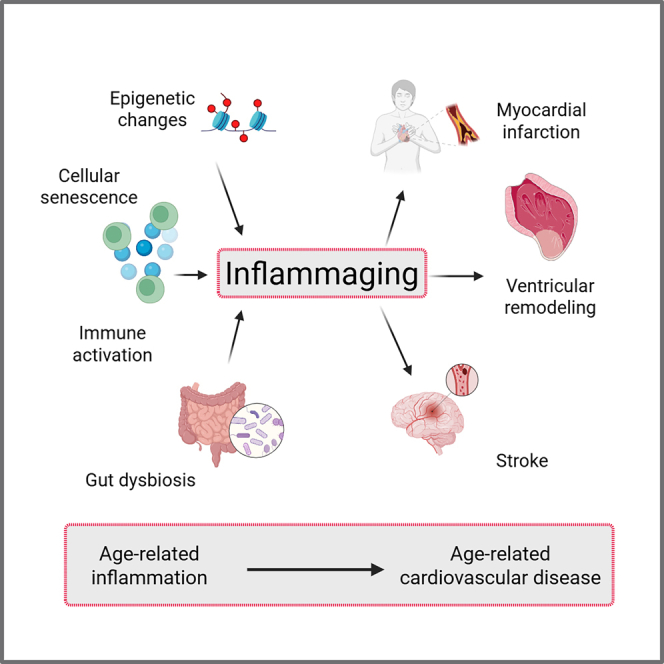

## Teaser

The complex interplay of aging, inflammation, and cardiovascular disease is explored in this review by Spray et al. Cellular senescence, mitochondrial dysfunction, epigenetic alterations, and the aging gut microbiome are explored, with discussion of clinical sequelae and novel treatment strategies.

## Introduction

The concept of inflammaging was introduced by Franceschi and colleagues in 2000, who used the network theory of aging to argue that repeated antigenic stimuli throughout life contributes to aging through a persistent stress response.^1^ This theory provided a framework for the well-known, apparently contradictory, observations in older people of (1) increased susceptibility to infections, suggesting a less active immune system,^2^ and (2) higher serum levels of cytokines and chemokines and higher incidences of autoimmune diseases, such as giant cell arteritis, suggesting an over-activated immune system.^3^ As humans age, continuously responding to antigenic stress can lead gradually to a higher background level of immune activation.^4^ In an example of pleiotropic antagonism, in which a phenomenon can be both advantageous and disadvantageous in different circumstances, this immune activation ultimately predisposes to inflammatory diseases in aging, including many cardiovascular (CV) diseases. CV disease (CVD) constitutes the largest causes of morbidity and mortality in people over 65 years old in the United States, with over 2 million deaths in this group annually.^5^ Understanding why the prevalence of CVD increases so greatly with age is a key research priority.^6^

The mechanisms by which inflammaging impacts the CV system are broad, and this review will cover hallmarks of CV aging including cellular senescence, dysregulation of leukocytes, mitochondrial dysfunction, epigenetic changes, and gut dysbiosis.^7^ These mechanisms predispose older people to the spectrum of CVD, including atherosclerosis of the heart and elsewhere, heart failure (HF), and thrombosis. Finally, we will explore therapeutic strategies, which target a variety of mechanisms of inflammaging, at various stages of translation, and demonstrate how manipulating the aging immune system may reduce the burden of CVD in older people.

## Mechanisms of CV inflammaging

### Cellular senescence and senescence-associated secretory phenotype

Cellular senescence was originally defined as a cell fate decision resulting in irreversible growth arrest, governed by tumor-suppressor pathways centered on p53/p21^CIP1^ and p16^INK4a^/Rb.^8^ However, this definition has since expanded to include a range of additional phenotypic features beyond cell-cycle arrest. These include mitochondrial dysfunction, cellular hypertrophy, altered morphology, disrupted proteostasis, and the development of a senescence-associated secretory phenotype (SASP).^9^^,^^10^ The SASP comprises a complex mix of pro-inflammatory cytokines, chemokines, growth factors, and proteases that can significantly reshape the surrounding tissue microenvironment.^9^ Importantly, SASP expression typically emerges after the establishment of growth arrest, often days following the initial stress signal, and is maintained by transcription factors such as nuclear factor κB (NF-κB) and CCAAT-enhancer-binding-protein beta (C/EBPβ).^11^ Recent findings have challenged the traditional view that senescence is restricted to proliferative cells, showing that post-mitotic cells such as cardiomyocytes (CMs) can also adopt a senescent-like state.^12^ This suggests that senescence can arise independently of cell division. In the heart—particularly within the predominantly quiescent myocardium—non-proliferative features such as mitochondrial dysfunction, hypertrophy, and SASP expression are now recognized as central contributors to senescence-driven tissue dysfunction, rather than the loss of replicative potential alone.

Although senescence is often viewed as a detrimental process that contributes to tissue dysfunction and age-related disease, it is, in fact, an evolutionarily conserved program with important beneficial roles, particularly in early life. During embryonic development, senescence occurs at defined anatomical sites to facilitate tissue patterning and organogenesis, after which senescent cells are efficiently cleared by macrophages as part of normal developmental remodeling.^13^ In adulthood, cellular senescence serves as a crucial tumor-suppressive mechanism by irreversibly arresting the proliferation of damaged or oncogene-activated cells, thereby preventing malignant transformation.^14^ However, the beneficial effects of senescence are context dependent and largely short lived. With age, senescent cells gradually accumulate in tissues due to impaired clearance, leading to chronic secretion of pro-inflammatory SASP factors.^15^ This contributes to inflammaging, tissue degeneration and the onset of age-related diseases, including CVD. This duality exemplifies antagonistic pleiotropy, where a mechanism that promotes organismal fitness early in life becomes detrimental in later years.^16^

Senescent cells are characteristically resistant to apoptosis due to the upregulation of pro-survival and anti-apoptotic genes, collectively referred to as senescence cell anti-apoptotic pathways (SCAPs), a property believed to also contribute to their accumulation and persistence with age. Transcriptomic analyses have shown that senescent cells overexpress anti-apoptotic proteins such as BCL-2 and BCL-xL and activate pro-survival pathways like phosphoinositide 3-kinase (PI3K)/Akt.^17^ This insight has led to the development of a class of compounds known as senolytics, which selectively target SCAPs to induce apoptosis in senescent cells while sparing healthy proliferative and quiescent cells.^17^ Senolytics not only hold therapeutic potential but have also been instrumental in demonstrating a causal role for senescent cell accumulation in the pathophysiology of various diseases, including CVD.

### Immune system aging and dysregulation

Increased myeloid differentiation from hematopoietic stem cells (HSCs), with reduced lymphopoiesis, has been identified as a hallmark of immune aging.^18^ In mice, although myeloid-biased HSCs are functionally similar from young and old donors, they persist in greater numbers than lymphoid-biased HSCs—leading to a pro-myeloid imbalance of hematopoiesis.^19^ Studies of young and old human bone marrow specimens have revealed that a similar myeloid bias occurs in human aging, showing this to be an evolutionarily conserved feature of aging.^20^ In the blood count, this can be observed as a raised neutrophil-to-lymphocyte (NLR) ratio, which is commonly seen in older individuals and appears to correlate with markers of frailty such as decreased grip strength.^21^ Raised NLR also predicts mortality, including CV mortality, in a range of clinical situations,^22^ although it is not clear whether the NLR is mechanistically important in determining survival or acts as a biomarker of advanced biological and immunological age.

In the heart, macrophages have a central role in regulating inflammation. CCR2− macrophages are embryonically derived, are self-sustaining, and appear to contribute to repair and the resolution of inflammation after myocardial injury, with upregulation of genes involved in myogenesis, epithelial-to-mesenchymal transition, and signal transducer and activator of transcription-5 (STAT5)/interleukin (IL)-2 signaling.^23^ CCR2+ macrophages, in contrast, are derived from circulating monocytes and have a pro-inflammatory phenotype.^23^ As individuals age, embryonically derived CCR2− macrophages are replaced with pro-inflammatory CCR2+ macrophages, which may directly lead to chronic inflammation in the myocardium.^24^

Adaptive immunity also undergoes significant alterations with advancing age. Most striking is the involution of the thymus, the organ responsible for a diverse and specific repertoire of T cell receptors (TCRs).^25^ Consequently, older adults have fewer naive T cells reaching the periphery and sustain T cell populations more heavily through clonal expansion of memory cells—reducing the diversity of TCRs. A second key feature of T cell aging is the accumulation of dysfunction mitochondria, largely due to defective clearing of aberrant mitochondria through autophagy.^25^ In one seminal experiment, mouse T cells were “aged” through knockout of mitochondrial transcription factor A. These cells switched to an inflammatory phenotype similar to that observed in older animals, and the mice developed aging-associated impairment in multiple organ systems.^26^ This included diastolic cardiac dysfunction and evidence of lung congestion—both features of the human disease HF with preserved ejection fraction (HFpEF). In another mouse model, CD8^+^ T cell depletion reduced atherosclerosis, and this was reversed by adoptive transfer of CD8^+^ T cells only from old, and not young, donors—suggesting again that T cell aging is pathogenic in CVD.^27^

It is also now understood that immunological memory is present in both the adaptive and innate immune systems, and in the latter it is termed *trained immunity* or *innate immune memory*.^28^ Through metabolic and epigenetic reprogramming, multiple CV risk factors induce trained immunity in the myeloid cell compartment.^29^ In experimental models, monocytes with a trained immunity phenotype aggravate atherosclerosis development, suggesting that trained myeloid cells may contribute to CVD in humans.^30^ In mice with subclinical endotoxemia, neutrophils too can shift to a persistent inflammatory state and accelerate atherosclerosis on adoptive transfer.^31^ Interestingly, a similar reprogramming of neutrophils occurs with an alternating high-fat diet, with greater IL-1β production and neutrophil infiltration of atherosclerotic plaques.^32^ Data on trained immunity in aging patients are scarce, but it may represent a key mechanism by which repeated pathogenic stimulus throughout life and the pro-myeloid bias of aging HSCs prime the immune system toward inflammation and CVD.

In summary, aging is associated with changes across the immune system in both mice and humans, and there are multiple mechanisms by which these changes can cause chronic, low-level inflammation and promote CVD.

### Mitochondrial dysfunction and oxidative stress

Mitochondria are responsible for the generation of adenosine triphosphate, act as a metabolic hub involved in myriad processes, and are critical signaling organelles in cellular homeostasis. Reactive oxygen species (ROSs) generated in mitochondria play a central role in various signaling pathways^33^ and are also relevant for disease. Under physiological conditions, mitochondria are considered the major source of ROS within cells. The electron transport chain involves multiple single electron transfer steps, which create superoxide anions as a by-product. This highly reactive molecule is rapidly converted to hydrogen peroxide by mitochondrial superoxide dismutase, and together these two species are considered mitochondrial ROS.

Mitochondrial dysfunction is one of the hallmarks of aging^34^ and is intricately involved in many age-associated diseases. Several factors have been demonstrated to trigger the decline of mitochondrial function during aging, including oxidative stress, oxidation of macromolecules, abnormal energy metabolism, and reduced mitochondrial biogenesis.^35^^,^^36^ This entails increased ROS production by the mitochondria themselves making them not only a target for but also a primary origin of oxidative stress. In addition, important quality control mechanisms in the mitochondria are impaired during the aging process and, under oxidative stress, mostly induced by damage to macromolecules involved in autophagy and mitophagy.^35^^,^^37^ This leads to the release of mitochondrial DNA and RNA into the cytosol, which is normally curtailed by mitophagy.^38^ The mitochondrial nucleic acids belong to the so-called damage-associated molecular patterns (DAMPs), which trigger the innate immune response inducing a sterile inflammation that activates mechanisms to remove abnormal intracellular components. Sustained sensing of DAMPs is detrimental as it leads to the release of inflammatory cytokines. Oxidative stress induces not only mitochondrial dysfunction but also cell death in various forms. This leads to the release of intracellular components, which then serve as DAMPs primarily for neighboring cells, but also for cells in distant tissues, and thus can induce systemic inflammation.^39^

Besides serving as DAMPs, mitochondrial components can directly trigger activation of the inflammasome. Peroxidation of the mitochondrial lipid cardiolipin leads to its translocation from the inner to the outer mitochondrial membrane, where it can directly bind and activate the NLRP3 inflammasome, leading to the production of pro-inflammatory cytokines.^40^ In summary, mitochondrial dysfunction is directly interconnected to systemic inflammation. Improvement of mitochondrial functionality in CVD may, therefore, reduce the inflammatory burden and improve outcomes. Multiple potential therapies have been tested with the goal of improving mitochondrial functionality in CVD, including the telomerase activator TA-65, caffeine, linoleic acid, and metformin. These substances show promise in improving not only mitochondrial function but also clinical condition after myocardial infarction (MI).^41^^,^^42^^,^^43^^,^^44^

### Epigenetic changes and dysregulated gene expression

Epigenetic modifications, which regulate gene expression without altering the DNA sequences, are a key driver of chronic low-grade inflammation in aging CV systems,^45^ influencing both health span and lifespan. Aging triggers sporadic epigenetic changes due to both endogenous and exogenous factors, which are closely linked to CVD risks.^46^ Sex-specific differences in DNA methylation patterns suggest a critical role in understanding disease phenotypes and tailoring sex-specific treatments.^47^ Accelerated epigenetic aging, a personalized predictor of biological age, has been linked to subclinical atherosclerosis through systemic inflammation.^48^ Since epigenetic mechanisms can be modified by pharmacological agents, lifestyle changes, and diet, epigenetic clocks tracking biological age hold promise for early interventions targeting biological aging.^49^

DNA methylation involves the addition or removal of methyl groups by DNA methyltransferases (DNMTs) and ten-eleven translocation dioxygenases (TETs), primarily targeting CpG sites. This dynamic process, regulated by genetic and environmental factors, influences inflammatory pathways in circulating leukocytes, cytokine release, and CVD progression.^50^ While most CpG sites are methylated in mammals, aging induces global DNA hypomethylation alongside localized hypermethylation, contributing to genomic instability.^51^ Atherosclerotic lesions exhibit global hypomethylation, while promoter regions of atheroprotective genes involved in vascular function are hypermethylated.^52^ Age-related hypomethylation in cytokine promoters further drives vascular aging and inflammation.^53^ The efficacy of IL-1β-targeting therapies like canakinumab may be influenced by DNA methylation patterns, explaining increased CV risks in patients with TET2 or DNMT3A mutations.^54^

Aging impacts histone levels and post-translational modifications, altering chromatin structure and accessibility, shifting from a tightly packed heterochromatin to loosely organized euchromatin. This transition contributes to genomic instability, loss of gene silencing, and increased retrotransposon activity^55^. Senescent cells accumulate senescence-associated heterochromatin foci, silencing cell-cycle genes like E2F target.^13^ Specific histone changes, such as reduced H3K9me3 and increased H4K20m33 levels, promote inflammaging.^56^

Non-coding RNAs, including microRNAs, long non-coding RNAs, and circular RNAs, play critical roles in aging and inflammation and are associated with all-cause mortality and age-related traits.^57^ Increased levels of miR-34 and reduced expression of its target gene, SIRT1, have been identified in replicative-senescent human vascular cells and aged mouse aortas^,^.^58^ In humans, miR-34 is associated with aortic stiffness, a surrogate marker of arterial aging, and the presence of CAD.^59^ miR-34 deletion in leukocytes reduces atherosclerosis and enhances Sirt1 expression.^59^ Finally, emerging evidence underscores the importance of RNA-binding proteins and RNA modifications in age-related diseases.^60^ Research into RNA metabolism’s role in inflammaging is expected to expand significantly in the coming years.

### Gut microbiome and systemic inflammation

**Figure 1:**
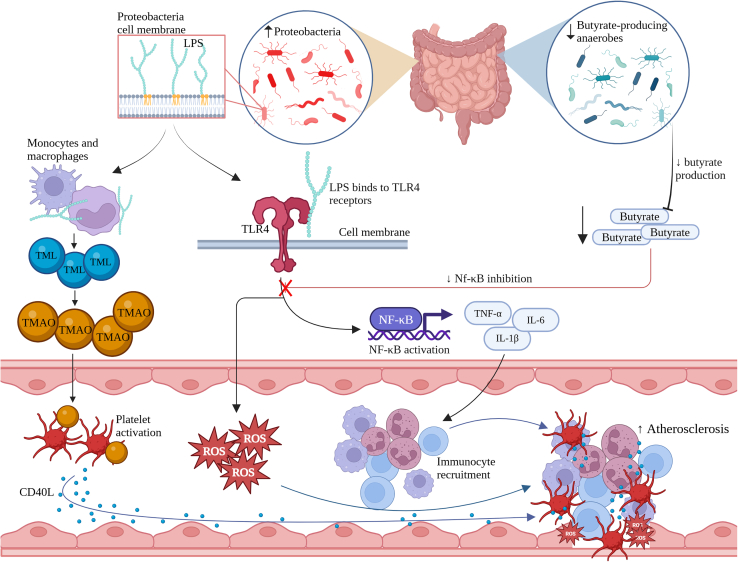
Age-related gut dysbiosis enhances systemic inflammation CD40L, cluster of differentiation 40 ligand; IL-1β, interleukin-1 beta; IL-6, interleukin 6; LPS, lipopolysaccharide; NF-κB, nuclear factor κB; ROS, reactive oxygen species; TLR4, Toll-like receptor 4; TMAO, trimethylamine N-oxide; TML, trimethyllsine; TNF-α, tumor necrosis factor alpha.

The human gastrointestinal tract hosts a large diversity of microorganisms. With aging, the gut microbiome loses this diversity and undergoes a change in the proportion of different microbes termed dysbiosis (Figure 1). The inflammatory consequences of this dysbiosis have been the subject of much study in recent years.^61^ Anaerobe species show particularly marked decline with aging, such as *Faecalibacterium prauznitzii* and *Clostridium cluster XIVa*—both of which exert anti-inflammatory effects.^62^ *F. prauznitzii* has been observed to inhibit IL-1β, causing reduced levels of IL-8 secretion and NF-κB activation,^63^ and both *Clostridium cluster XIVa* and *F. prauznitzii* produce high levels of butyrate,^64^ which reduces lipopolysaccharide (LPS)-induced expression of cytokines by inhibiting NF-κB activation.^65^ *Proteobacteria*, in contrast, increase with increasing age.^62^ The outer cell membrane of these gram-negative bacteria comprises LPSs, which potently activate the immune system through Toll-like receptor (TLR)-4- and the MYD88-dependent pathway,^66^ with consequent elevation of CV risk. LPS, which is more abundant in the aging gut, also increases the production of trimethyllysine,^67^ a trimethylamine N-oxide precursor, which acts as an agonist on the platelet membrane, causing platelet hyperactivation.^68^ Platelet hyperactivation releases CD40 ligand, triggering an inflammatory response in the endothelium that contributes to further endothelial dysfunction. CVD itself can further exacerbate gut dysbiosis in a feedback loop, with HF patients displaying lower levels of butyrate-producing bacteria.^69^ In summary, age-related changes in gut flora contribute to a systemic inflammatory milieu and are tightly linked to CVD pathogenesis and progression.

## Consequences of CV inflammaging

### Atherosclerosis development and plaque instability

There is an inextricable link between aging, the development of atherosclerosis, and its consequence in triggering acute coronary syndromes (ACSs).^70^ The almost exponential rise in the frequency of ACS in individuals between 30 and 70 years of age is testament to the accelerating development and destabilization of atherosclerotic plaques leading to atherothrombosis and MI or stroke. The prevailing theory that atherosclerosis simply results from an accrual of lipid and arterial damage over time has been complimented by an appreciation that aging-related processes contribute to plaque development in which a gradual loss of homeostasis results in impaired immunity, perturbed metabolism, and declining regenerative capacity.^71^ The relationship between aging and the acceleration of plaque development and destabilization is complex as aging not only accelerates atherosclerosis but also promotes the comorbid risk factors that contribute to atherosclerotic plaque development including type 2 diabetes, hypertension, chronic kidney disease, and liver dysfunction and fibrosis.^72^

Atherosclerosis is primed by endothelial dysfunction, which limits the early stages of disease to sites of disturbed flow, especially in males.^73^ Clinically, endothelial dysfunction is defined as an impairment of endothelial-dependent vasorelaxation, while at a cellular level it is recognized as a reduction of nitric oxide (NO) bioavailability, increase in oxidative stress, and enhanced activation of NF-κB, leading to increased expression of inflammatory cytokines and adhesion molecules and a reduced anti-thrombotic capacity of the endothelium.^74^^,^^75^ The reduction of endothelial-derived NO, a significant regulator of smooth muscle function, inhibiting proliferation and migration,^76^ may play a dominant role in allowing the escape of limited numbers of arterial smooth muscle cells from the media, migrating to the intima and forming a smooth muscle cell-rich intimal hyperplasia, the seedbed of human atherosclerosis.^77^ Resultant matrix accumulation within the intima traps low-density lipoprotein (LDL), allowing its oxidation and recognition as a DAMP by TLRs triggering inflammation and immune cell recruitment.^78^^,^^79^ It is the acceleration of this process with aging that overwhelms the ability of cells to efflux excess cholesterol from the intimal thickening and results in the accumulation of extracellular lipid within the now “atherosclerotic” plaque.^77^

Aging plays a role in all stages of atherosclerotic plaque development,^74^ with increased endothelial cell dysfunction, ROS production—quenching NO and modifying LDL, NF-κB activation, smooth muscle cell (SMC) proliferation, expansion of the intimal hyperplasia with synthetic smooth muscle cells laying down disorganized matrix that traps LDL, and matrix modification with advanced glycation end products. The aging immune system plays a key role in the acceleration of atherosclerosis.^80^ As already discussed, aging of the bone marrow niche and stem cell aging^81^ results in alteration of the distribution of monocyte subsets, monocyte-neutrophil ratios, and platelet production, all of which are well established risk factors for ACS through increasing the inflammatory environment within the plaque. The aging-related contribution of clonal hematopoiesis with indeterminate potential,^82^ which also modifies immune cell function, has been shown to increase ACS prevalence^83^ again through shifting the inflammatory response within the plaque, promoting cap thinning and the development of rupture-prone thin-fibrous capped atheroma.^84^ Within the later stages of plaque evolution, SMC and macrophage senescence reduce fibrous cap maintenance, promote cap thinning, and contribute to plaque inflammation through secretion of SASP-related factors.^85^

The accumulation of senescent cells has also been implicated in the pathogenesis and progression of atherosclerosis. In murine models of atherosclerosis, such as *Ldl*^*r−/−*^ mice fed a high-fat diet, senescent cells have been detected within plaque-rich regions of the aortic arch, marked by elevated expression of p16^Ink4a^ and SASP components including MMP-3, MMP-13, IL-1α, and tumor necrosis factor alpha (TNF-α).^86^ Moreover, in *Ldlr*^−/−^ mice, treatment with the senolytic navitoclax, after senescence had already been established, significantly reduced senescent cell burden, decreased plaque size and number, and lowered levels of pro-atherogenic mediators such as monocyte chemoattractant protein-1 (MCP-1), IL-1α, TNF-α, and vascular cell adhesion protein-1 (VCAM-1).^86^ Complementary studies in *ApoE*^−/−^ mice treated with a senolytic cocktail of dasatinib and quercetin (D + Q) demonstrated reductions in senescent cell markers and plaque calcification, though without a substantial change in plaque size.^87^ These effects have been further validated using transgenic mouse models that enable selective clearance of senescent cells, providing strong causal evidence for the role of cellular senescence in atherogenesis.^86^

The role of aging in plaque rupture and resultant atherothrombosis are largely the consequences of the processes mentioned earlier. Biomechanical failure of the fibrous cap that leads to plaque rupture is promoted by SMC apoptosis and senescence preventing the replenishment of collagen within the fibrous cap.^88^ Inflammation, and also potentially macrophage senescence through upregulation of SASP, increase the production of matrix metalloproteinases,^89^ promoting degradation of the fibrous cap and further degradation of the structural support of the fibrous cap, eventually leading to mechanical failure and rupture. The triggers of plaque erosion are more elusive, likely resulting from a multifactorial stress response reducing autophagy thus limiting adhesion.^90^ Cigarette smoking is an identified risk factor for plaque erosion^91^ and accelerates biological aging through an Nrf2-promoted stress response.^92^ The capacity of Nrf2-responsive OSGIN1 and OSGIN2 to mediate and coordinate cellular stress resulting in endothelial detachment has identified a potential role for aging accelerating stressors in promoting plaque erosion.^90^

### Endothelial dysfunction and vascular stiffness

Increased oxidative stress is a common denominator within aging-related pathology, driving cellular dysfunction and senescence while also increasing many pro-atherogenic processes.^93^ Oxidative stress is especially important in driving endothelial dysfunction, as an activator of NF-κB^74^ and through the quenching of NO resulting in the creation of the highly reactive peroxynitrite free radical.^94^ Oxidative stress increases with aging and is also induced by many pro-atherogenic risk factors including poor diet, sedentary behavior, smoking, diabetes, hyperlipidemia, chronic kidney disease, and hypertension, reinforcing the link between atherosclerosis and aging.^72^^,^^95^

As previously discussed, quenching of NO by prooxidant free radicals provides a permissive environment for smooth muscle cell proliferation, migration, and expansion of the intima. Additionally, prosenescent changes within the endothelium reduce the bioavailability of NO and result in a reduced capacity to repair.^96^ Limited endothelial denudation has been observed overlying human atherosclerotic plaques and resultant platelet adhesion.^97^ Platelet binding and release of growth factors such as platelet-derived growth factor and fibroblast growth factor further promote smooth muscle cell phenotypic shift, proliferation, and matrix production.^98^^,^^99^ These combined effects result in the development of a diffuse intimal hyperplasia with aging, dysregulated production of collagenous matrix by synthetic smooth muscle cells that not only promotes pro-atherogenic lipid retention but also results in arterial stiffening. Concomitant with this, aging associates with an increase of elastin and collagen crosslinking by both enzymatic action and oxidation and modification by advanced glycation end products.^100^ As collagen turnover is a very slow process, the passage of time results in the accumulation of more densely cross-linked, less elastic matrix, which itself affects cell function,^101^ promoting both endothelial^102^ and smooth muscle cell dysfunction and senescence,^103^ establishing a positive feedback loop that is likely to accelerate with aging.

Cellular plasticity may also play a role in aging-related vascular remodeling, through additional contribution to intimal hyperplasia and matrix production. The role of synthetic smooth muscle cells in forming the intimal hyperplasia is well established,^104^ but more recently the potential for endothelial-to-mesenchymal transition,^105^ particularly at sites of disturbed flow, has also highlighted and reinforced the concept that the intima may be derived from cells from multiple origins including macrophage and endothelial cells along with smooth muscle cells, with their relative contribution to vascular stiffening, vascular dysfunction, and atherosclerosis in human disease remaining an area of active research.

### Cardiac remodeling and heart failure

HF is a major public health problem, with high rates of hospitalization and death despite advances in medical therapy. Inflammation, both within the myocardium and systemically, is a key driver of adverse left ventricular remodeling in both ischemic and non-ischemic HF.^106^ Three large trials of anti-cytokine drugs in patients with HFrEF have produced negative results,^107^^,^^108^ but seminal studies showing the importance of lymphocytes in HF models provide a new route forward.

After left anterior desending artery (LAD) ligation, the development of ischemic HF is attenuated in mice without CD4^+^ T cells,^109^ and transfer of CD4^+^ T cells from mice that have undergone LAD ligation induces an HF-like syndrome in recipient, naive mice.^110^ Similar experiments in the transverse aortic constriction model have yielded similar results.^111^ Remarkably, regulatory T cells, critical for ordered healing at the time of MI, develop a pro-inflammatory phenotype during chronic HF, becoming essential for disease progression.^112^ In summary, T lymphocytes are both necessary and sufficient to induce HF in mice, and in the chronic setting even regulatory T cells become inflammatory and pathogenic.

Much previous research has focused on HFrEF, but it is HFpEF, which most appears to be a disease of aging.^113^ Animal models of HFpEF, although harder to create than those of HFrEF, also support a role for immune cells.^113^ The combination of a high-fat diet and induced hypertension causes a HFpEF-like phenotype in mice, but not in T cell-deficient mice,^114^ and withdrawal of these factors reversed deficiencies in T cell activation and structural cardiac changes. This suggests that some immunological and cardiac structural changes in HFpEF may be reversible with targeted treatment of underlying comorbidities.

### Thrombosis and coagulation changes

Conditions associated with chronic inflammation are associated with an increased risk of thrombosis and CV events.^115^ Chronic exposure to cytokines including IL-1β, IL-6, TNF-α, and C-reactive protein (CRP) promotes endothelial dysfunction with increased expression of surface adhesion molecules (VCAM-1, ICAM-1, and selectins),^116^ facilitating leukocyte adhesion, activation, and neutrophil extracellular trap formation (NETosis, the release of chromatin nets that can trigger thrombosis).^117^ Loss of endothelial barrier function (reduced vascular endothelial (VE)-Cadherin expression), extracellular matrix exposure, and release of von Willebrand factor (VWF) combined with reduced NO production promote platelet adhesion and platelet activation. Furthermore changes to endothelial expression of coagulation activators and inhibitors increase the thrombin-generating potential of the plasma.

Both inflammation and aging are associated with increased platelet hyperreactivity. Prolonged exposure to TNF-α, IL-6, and IL-1β, in both the plasma and the bone marrow, primes platelets for activation, aggregation,^118^ and secretion of pro-coagulants^119^ and release of microvesicles.^120^ Activated platelets also recruit neutrophils, exacerbating the inflammation. Upregulation of tissue factor on endothelial cells and monocytes, coagulation factors, thrombin generation, and fibrinogen all promote fibrin clot formation, independent of platelet activation.^121^ Chronic inflammation also increases circulating plasminogen activator inhibitor-1 (PAI-1) and thrombin activatable fibrinolysis inhibitor, impairing fibrinolysis and enhancing clot stability.^122^

**Figure 2:**
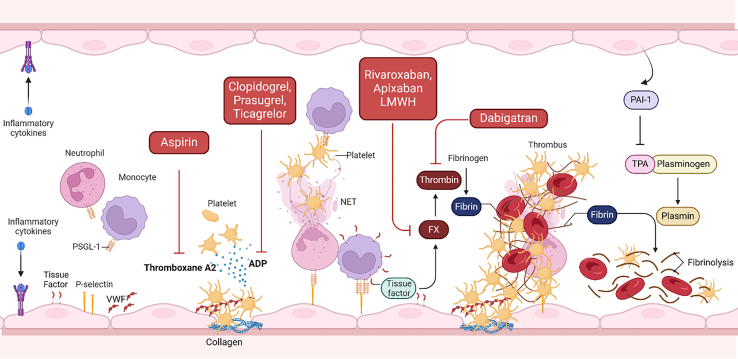
Inflammation predisposing to thrombus formation and target pathways of commonly used anti-thrombotic drugs

Over time, these effects of inflammaging lead to a hemostatic imbalance and prothrombotic state, increasing the risk of MI or stroke, and, in clinical studies, markers of thrombosis (soluble P-selectin, PAI-1, and VWF)^123^ are predictive of adverse CV outcomes.^124^^,^^125^ Current anti-thrombotics targeting platelet activation or coagulation also exhibit anti-inflammatory effects, demonstrating a two-way crosstalk between inflammation and thrombosis. Rivaroxaban, a factor Xa inhibitor, reduces atherosclerosis, ischemic events,^126^ and inflammation by attenuating NF-κB and inflammasome activity and lowering IL-6 and CRP levels.^127^ Similarly, heparin can disrupt the process of NETosis; antiplatelet drugs, such as the P2Y12 antagonist ticagrelor, reduce inflammatory cytokines; and aspirin, through cyclooxygenase inhibition, reduces inflammatory prostaglandin and thromboxane A2 synthesis.^128^^,^^129^^,^^130^ Current anti-thrombotic therapies are often offset by bleeding complications, and developing safer anti-thrombotics is an unmet clinical need. Current successes with glycoprotein 6 (GPVI) antagonists look promising,^131^ as do preclinical studies targeting the VWF/ADAMTS13 axis.^132^ Further advancements in our mechanistic understanding of immunothrombosis (summarized in Figure 2), however, are needed to develop targeted anti-inflammatory interventions and mitigate the thrombotic and CV risks associated with inflammaging.

## Biomarkers of CV inflammaging

### Circulating biomarkers

CRP, IL-6, and fibrinogen are well studied in the association of inflammation with CV aging.^1^^,^^2^ CRP is a biomarker for the IL-1β/IL-6/CRP axis and is a robust predictor of atherosclerotic events.^133^ IL-6 has also been implicated in the progression of carotid atherosclerosis, by increasing vascular permeability and promoting monocyte infiltration into the intima, and in coronary artery calcium scores.^134^^,^^135^ Notably, Mendelian randomization studies have showed that genetic variances involving IL-6, but not CRP, were associated with lower lifetime levels of vascular risk.^136^ This suggests that IL-6 may have a causative role in CVD, while CRP serves as a widely available biomarker of the IL-1β/IL-6 axis. Further, IL-6 regulates the synthesis of fibrinogen, a glycoprotein biomarker implicated in aging CVD mainly through the activation of platelets via GP IIb/IIIa receptors, the increased expression of intercellular adhesion molecule-1 (ICAM-1), and the production of vasoactive factors by the endothelial cells.^137^

Amyloid-beta 1-40 peptide is increasingly recognized as a key mediator of inflammaging in circulating leukocytes and the vascular wall.^138^^,^^139^ In experimental studies, this aging-induced peptide exerts pro-inflammatory effects, stimulating immune cells to release cytokines and ROSs.^138^^,^^139^ Such chronic low-grade inflammation accelerates cellular senescence, impairing leukocyte function and contributing to vascular stiffness.^138^^,^^139^ Amyloid-beta 1-40 also disrupts vascular homeostasis, promoting atherosclerotic changes and compromised vascular repair mechanisms.^140^^,^^141^ As a result, its elevated levels are linked with systemic inflammation and CV dysfunction, ultimately increasing susceptibility to age-associated CVD and systemic inflammatory conditions. As a biomarker, measuring circulating amyloid-beta 1-40 may improve early detection and risk stratification of heart disease patients at risk for future major adverse CV events, helping clinicians identify patients at higher risk for a poorer prognosis.^49^ Further research is needed to validate the clinical value of circulating amyloid-beta as an inflammaging marker in age-related diseases.

The complex interplay between pro- and anti-inflammatory markers in inflammaging underscores the concept that an array of inflammatory biomarkers, rather than a single one, holds value for characterizing and assessing CVD risk. Circulating markers like CRP, IL-6, and fibrinogen enhance CVD risk assessment by providing insights beyond established traditional factors. From a primary prevention perspective, a level of high-sensitivity CRP ≥ 2.0 mg/L is suggested as a CV risk factor by the American College of Cardiology/American Heart Association for CVD prevention.^142^ The Reynolds risk score also utilizes high-sensitivity CRP to estimate the CVD risk in women aged over 45, guiding further medical management.^143^ In secondary prevention, biomarkers such as CRP, IL-1β, and IL-6 highlight the residual risk in patients already receiving treatment, with the Justification for the Use of Statins in Prevention: an Intervention Trial Evaluating Rosuvastatin (JUPITER) and Canakinumab Antiinflammatory Thrombosis Outcome Study (CANTOS) studies emphasizing their role in identifying individuals who may benefit from targeted interventions.^144^ These findings emphasize the potential of inflammatory biomarkers in precision medicine, refining CVD risk assessment and guiding targeted interventions.

### Imaging biomarkers

**Figure 3:**
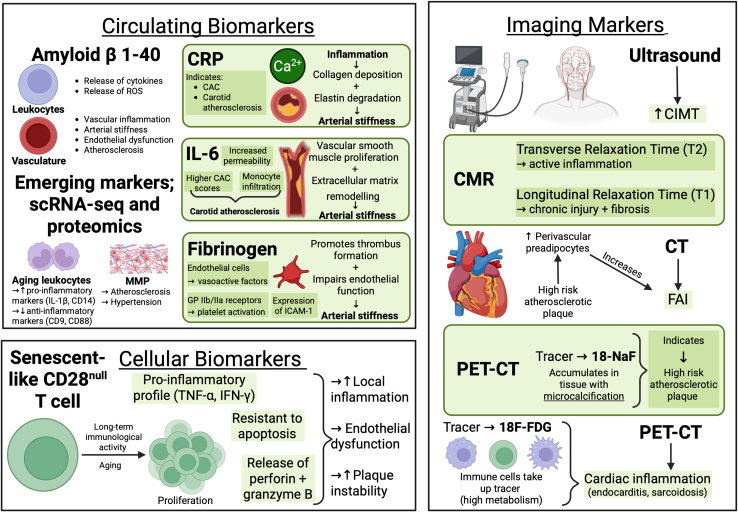
Markers available to assess cardiovascular inflammaging ROS, reactive oxygen species; CAC, coronary artery calcium scores; scRNA-seq, single-cell RNA sequencing; MMP, matrix metalloproteinase; CIMT, carotid intima-medial thickness; CT, computed tomography; FAI, fat attenuation index; PET-CT, positron emission tomography CT; CMR, cardiovascular magnetic resonance.

CV inflammation can be detected, directly or indirectly, with diverse imaging modalities including ultrasonography, cardiovascular magnetic resonance (CMR), computed tomography (CT), and radionuclide imaging (Figure 3). Most non-invasively, ultrasound allows the measurement of the thickness of the intima and media of the carotid artery—the carotid intima-medial thickness (CIMT)—which is a measure of subclinical atherosclerosis.^145^ It may also give an indirect indication of systemic inflammation, as CIMT has been shown to correlate with inflammatory markers.^146^

CMR is often the first-line modality for assessment of cardiac inflammation due to its accessibility, lack of ionizing radiation, and unparalleled contrast resolution. Active inflammation is best visualized through mapping of transverse relaxation time (T2), with raised T2 either globally or in a specific region, being a histologically validated surrogate marker of myocardial edema. Longitudinal relaxation time (T1) mapping is less specific for inflammation but more sensitive to chronic injury and fibrosis, for which inflammation is one possible cause. The use of gadolinium-based contrast agents can show specific patterns of late-gadolinium enhancement, suggesting different etiologies.^147^ CMR-detected myocardial inflammation and fibrosis have also been described outside of myocarditis and dilated cardiomyopathy, with one landmark study finding higher native T1 time in patients with long COVID.^148^

The excellent spatial and temporal resolution of CT imaging make it first-line for non-invasively imaging the coronary arteries and also allows assessment of perivascular adipose tissue. When next to a high-risk atherosclerotic plaque (HRP), pre-adipocytes are prevented from developing into larger, lipid-rich adipocytes through peroxisome proliferator–activated receptor (PPAR)-γ signaling.^149^ Adipose tissue adjacent to HRPs is therefore composed of smaller, lipid-poor cells, quantified on CT as a higher fat attenuation index (FAI), and prospective studies have found the FAI to be a strong predictor of CV mortality.^150^

Radionuclide imaging is also indispensable in imaging CV inflammation. The radionuclide tracer 18F-fluorodeoxyglucose (18F-FDG) is a glucose analog, which accumulates in cells with high metabolic glucose utilization. Immune cells, particularly macrophages, in inflamed tissues have high glucose metabolism, and 18F-FDG uptake therefore functions as a marker of tissue inflammation. 18F-FDG positron emission tomography (PET)-CT is the gold-standard imaging modality for assessment of cardiac inflammation, with European guidelines recommending 18F-FDG PET-CT in the diagnosis of cardiac sarcoidosis.^151^

Beyond these overtly inflammatory diseases, PET-CT shows promise in identifying HRP, using the tracer 18F-NaF, which localizes to tissues with micro-calcification, a marker of HRP.^152^ In The Prediction of Recurrent Events with 18F-Fluoride to Identify Ruptured and High-risk Coronary Artery Plaques in Patients with Myocardial Infarction study, patients with 18F-NaF avid coronary plaque had a nearly 2-fold risk of MI or CV death at 4 years, although the primary composite outcome including revascularizations was not significantly different.^153^ The use of PET-CT imaging to identify plaques at high risk of rupture, and initiate aggressive therapy earlier, is a promising future application.

### Cellular biomarkers/CD28^null^ T lymphocytes

Senescent-like CD28^null^ T lymphocytes (T cells) are an immune cell subtype that contributes significantly to CV inflammaging by sustaining tissue damage and maladaptive inflammation. The costimulatory molecule CD28, which is essential for strong immune responses, is absent from senescent CD28^null^ T cells. Age and long-term immunological activity, such as in atherosclerosis, cause these cells to proliferate.^154^ These are primarily cytotoxic CD8^+^ T cells, although a smaller subset of CD4^+^ CD28^null^ T cells also appears as people age. CD28^null^ T cells display decreased proliferation, a pro-inflammatory cytokine profile (including TNF-α and interferon [IFN]-γ), and resistance to apoptosis.^155^ These cells penetrate vascular lesions, intensify local inflammation and endothelial dysfunction, and increase plaque instability through release of the cytotoxic molecules perforin and granzyme B. Patients with ACS have elevated levels of IFN-γ-producing CD4^**+**^ T cells when compared to those with stable disease or healthy controls.^154^ CD4^**+**^CD28^null^ T cells differ dramatically in these patients, as they express significantly higher levels of OX40 and 4-1BB costimulatory receptors when compared to classical CD4^**+**^CD28^null^ T cells.^155^ We and others have shown that these cells typically express high levels of the fractalkine (CXCL10) receptor CX_3_CR1.^156^^,^^157^ Patients without expansion of the CD28^null^ T cell subset show <2% circulating CD28^null^ T cells out of all CD4^+^ T cells in peripheral blood.^158^ Blocking of the latter 2 receptors reduces the production of IFN-γ by the CD4 T cell subset, suggesting that antigens present in atherosclerotic plaques contribute to CD4 T cell responses and enhance inflammation, coinciding with a higher proportion of these cells in cytomegalovirus (CMV)-seropositive patients. A recent study showed that IL-7 and IL-15 drive the expansion and function of CD28^null^ T cells from ACS patients suggesting that IL-7/IL-15 blockade may prevent expansion of these cells and improve patient outcomes.^158^

## Therapeutic interventions targeting CV inflammaging

### Anti-inflammatory drugs

The theory that drugs exerting anti-inflammatory effects may benefit the CV system is not new, although historic trials, such as those of TNF-α inhibitors in HF, have been disappointing.^107^^,^^108^ One source of encouragement has been the understanding that statins have multiple beneficial effects in the CV system beyond plasma LDL cholesterol lowering. These effects include a reduction in oxidative and pro-inflammatory burden^159^ and improved CV outcomes even in the absence of hyperlipidemia,^144^ perhaps due to these anti-inflammatory effects.

Three seminal randomised controlled trials—CANTOS,^160^ the Colchicine Cardiovascular Outcomes Trial (COLCOT), ^161^ and the The Low-dose Colchicine 2 (LoDoCo2)^162^—have demonstrated the efficacy of targeting inflammation for secondary prevention of CV events.^49^ It is noteworthy that it was not until 2017, when the results of the CANTOS trial were published, that the inflammatory hypothesis of atherogenesis in humans was confirmed, and that there was proof of concept that addressing inflammatory risk in patients with CVD could prevent recurrent CV events. For secondary prevention, CANTOS tested canakinumab, an injectable antibody that inhibits the action of the inflammatory cytokine IL-1β, in individuals who had experienced a prior coronary incident. Canakinumab led to a 15% reduction in recurrent CV events, a significant decrease in high-sensitivity CRP levels, and surprisingly a decreased incidence of cancer.^160^ Unfortunately, there was also a higher incidence of infections and sepsis, limiting its translational potential.

A meta-analysis of six randomized clinical trials involving nearly 15,000 patients with prior coronary disease demonstrated a consistent benefit of colchicine for the prevention of major adverse CV events.^163^ These results have led to colchicine, an anti-inflammatory medication, being approved for use in the United States to lower CV events in people with multiple CV risk factors or atherosclerosis, although the negative randomized controlled trial of colchicine and spironolactone in patients with ST elevation myocardial infarction (CLEAR) trial suggests colchicine may not provide benefit immediately after MI.^164^

Upstream regulators of inflammation, such as mitochondrial function, are also a promising target for immunomodulating therapies in CVD. In a randomized clinical trial in 90 patients with MI aged >65 years, treatment with the mitochondrial telomerase activator TA-65 reduced circulating inflammatory markers and increased the numbers of adaptive immune cells compared with placebo.^42^

Emerging studies on cell-based therapeutics suggest the potential for more precise interventions compared to traditional compound-based approaches. Notably, novel strategies involving the *ex vivo* reprogramming of resolving leukocytes—particularly neutrophils and monocytes—are under investigation. When reinfused, these reprogrammed cells may actively promote the resolution of vascular inflammation and attenuate atherosclerotic disease.^165^^,^^166^^,^^167^

### Senolytics and senomorphics

The two major therapeutic strategies being actively investigated as interventions for cellular senescence, and the impact of the SASP on CVD, are senolytics and senomorphics. Senolytics are a class of drugs that selectively eliminate senescent cells by exploiting their dependence on pro-survival pathways, known as SCAPs.^168^ These pathways include BCL-2 family proteins, PI3K signaling, and PAI-1/2, which senescent cells upregulate to evade apoptosis.^17^^,^^169^ By targeting these mechanisms, senolytics effectively induce apoptosis in senescent cells while sparing normal proliferating and quiescent cells.

The first identified senolytic combination—dasatinib (D) and quercetin (Q)—remains the most widely studied.^17^ Dasatinib, a tyrosine kinase inhibitor, disrupts ephrin-dependent pro-survival signaling, while quercetin, a natural flavonol, inhibits PI3K and other survival pathways.^17^^,^^170^ In preclinical studies, D + Q has been shown to reduce senescent cell burden, alleviate myocardial fibrosis, and improve vascular function in murine models of age-related cardiac dysfunction and atherosclerosis.^17^^,^^87^ Similarly, navitoclax (ABT-263), a B-cell-lympoma (BCL)-2/BCL-XL inhibitor, effectively clears senescent cells, reducing myocardial hypertrophy and improving cardiac function in experimental models of age-related HF^171^ and ischemic injury.^172^

Beyond these, additional senolytic compounds—including cardiac glycosides (digoxin and digitoxin), HSP90 inhibitors, and p53-targeting molecules—have demonstrated broad-spectrum senolytic activity.^173^ However, concerns regarding potential on-target and off-target toxicities may hinder clinical translation. For instance, thrombocytopenia is the dose-limiting toxicity of navitoclax, arising from its inhibition of Bcl-XL in platelets.^174^ Dasatinib, meanwhile, is associated with cardiotoxic effects, including QT interval prolongation, arrhythmia, and palpitations.^175^ Rare but severe cases of congestive HF and ventricular dysfunction have also been reported with extended dasatinib use.^176^ Furthermore, given the heart’s limited regenerative potential and the strong association between CM loss and HF, the apoptotic mechanism of senolytics poses a challenge. Inducing apoptosis in senescent post-mitotic cell populations, specifically CMs, could exacerbate tissue damage and impede long-term recovery, complicating their therapeutic application.

Therefore senomorphics (or senostatics), which act by modulating the SASP, reducing inflammatory cytokine secretion without inducing apoptosis, may offer an alternative therapeutic approach. Several existing drugs have been repurposed as senomorphics due to their ability to suppress SASP-associated inflammation. Metformin, a widely used adenosine monophosphate-activated protein kinase (AMPK) activator used to treat type 2 diabetes, reduces SASP expression by inhibiting NF-κB signaling and IL-6 secretion.^177^ Preclinical and clinical studies suggest that metformin treatment attenuates myocardial remodeling, improves endothelial function, and reduces vascular inflammation, effects that may be partly attributed to its senomorphic properties.^177^ Ongoing trials, such as theTargeting Aging with Metformin study, aims to clarify metformin’s broader anti-aging potential.^178^ Similarly rapamycin, an mTORC1 inhibitor, has documented immunosuppressive properties, reduces SASP expression, and extends lifespan in preclinical models.^179^ In a murine model, rapamycin treatment decreased age-related CM hypertrophy, reduced interstitial fibrosis, and improved cardiac function, supporting its potential as a senomorphic therapy for CVD.^180^

Senescence-associated mitochondrial dysfunction can also be reversed by targeting senescence.^172^ Impaired mitophagy, a key cellular quality control mechanism, contributes to the accumulation of dysfunctional mitochondria in senescent cells. Notably, both metformin and rapamycin have been shown to stimulate mitophagy, which may underlie their senomorphic effects. In line with this, recent studies have demonstrated that senescent cells, including fibroblasts and primary human cardiac microvascular endothelial cells, exhibit reduced basal mitophagy, leading to the accumulation of dysfunctional mitochondria.^181^ Interestingly, silencing key mitophagy regulators such as PINK1, Parkin, or p62 via short hairpin RNA is sufficient to trigger cellular senescence. Notably, the small molecule STOCK1N, which binds with high affinity to the ZZ domain of p62—a critical regulator of mitophagy—can prevent senescence-associated mitophagy suppression and exerts senoprotective effects following cellular irradiation.^181^ Moreover, STOCK1N treatment in fibroblasts derived from older donors successfully rescues the senescent phenotype, suggesting that its senomorphic properties are independent of the initial senescence-inducing stimulus.^181^ Together, these findings highlight specific p62 activation as a promising therapeutic strategy to counteract the detrimental effects of senescence without inducing cell death.

### Lifestyle interventions

Exercise, diet, and sleep patterns are all significant determinants of CV health.^182^^,^^183^ Exercise’s beneficial benefits on the CV system can be partially attributed to its ability to raise plasma high-density lipoprotein levels, lower LDL levels, and improve insulin sensitivity,^184^ although it has wider effects on the aging immune system. Frequent exercise promotes mitochondrial biogenesis in the skeletal muscle and reduces systemic inflammation, which ultimately improves energy generation and slows the aging process of the heart and skeleton.^184^ When compared to a normal Western-style diet, the Mediterranean diet has been linked to lower CV risk, a lower incidence of major CV events in people with high CV risk, and protection against the progression of CVD following a major CV event.^185^ Randomized controlled trials have shown that, compared to calorie restriction or an *ad libitum* diet, intermittent fasting may improve health outcomes for people who are overweight or obese.^186^ An alternative approach to replicate the positive impacts of calorie restriction on lifespan is to follow a ketogenic diet. They are distinguished by a varied global calorie restriction and a restriction of carbohydrate intake (less than 20–30 g per day). This causes a change in energy metabolism from the ingestion of carbohydrates to triglycerides, which ultimately results in the creation of ketone bodies. When male old mice were fed an isocaloric ketogenic diet continuously, their median lifetime rose and their physiological functions were preserved.^187^ Meta-analyses of research on diabetic individuals, however, did not show that ketogenic diets offered any advantages beyond weight loss, currently limiting the translation of these encouraging findings.^188^

### Gut microbiome modulation

Several interventions can be done to modulate and change the gut microbiome, including probiotics, prebiotics, and fecal microbiome transplantation (FMT). Probiotics consist of living microorganisms that increase the quantity of beneficial bacteria, such as *Lactobacillus* and *Eubacterium*, and decrease the quantity of potentially harmful bacteria, such as *Enterococcus* and *Pseudomonas*.^189^ Prebiotics, in contrast, encourage the growth of beneficial bacteria by selectively feeding them.^190^ Prebiotics can also prevent the attachment of pathogenic bacteria to the intestinal cells and increase butyrate levels.^191^ It has also recently been reported that a synbiotic therapy, combining a prebiotic fiber and a *Lactobacillus* mucosa-rich diet, can significantly reduce intestinal and cardiac inflammation in porcine model of cardiometabolic disease and HFpEF.^192^ The treatment improved gut barrier integrity—evidenced by a 2-fold increase in mucosal thickness—and decreased pro-inflammatory immune cell infiltration in both the intestine and the heart. These findings suggest that synbiotics may offer a promising strategy to modulate gut-heart interactions and alleviate cardiac remodeling and inflammation in age-related CVD. Finally, FMT introduces an entire healthy microbiota from healthy donors and is an effective treatment for dysbiosis and dysbiosis-related conditions, such as *Clostridium difficile* infection.^193^ In a murine model of HFpEF, FMT induced improvements in both systolic and, importantly, diastolic function.^194^ Although promising, further work is needed to establish if these findings will translate to manipulation of the microbiome causing improvements in human CVD.

## Conclusion: Inflammaging as an opportunity for personalized medicine

Currently, CV risk assessment and management are largely based on conventional risk factors, such as the presence or absence of diabetes or level of LDL cholesterol.^195^ This relatively crude risk classification lags behind other areas of medicine, such as cancer care, and poses three problems to physicians treating older patients with or at risk of CVD. Firstly, in some patient groups, for example, those without detectable coronary calcium on CT imaging, standard primary prevention strategies with statins do not appear to confer a benefit.^196^ Secondly, atherosclerosis and its complications frequently occur in people without traditionally identifiable risk factors.^197^ Thirdly, older patients are at higher risk of adverse effects from some primary prevention medication, such as blood pressure (BP)-lowering agents.^198^ These observations call for a more personalized, patient-specific approach to risk reduction, especially in older people, and the emerging data from studies of inflammaging suggest key new determinants of risk. Biomarkers of CV inflammation may better risk-stratify patients and help guide therapy, while our advancing understanding of the mechanisms behind this inflammation may provide entirely new strategies for treatment. By deeply immunophenotyping patients, we may one day be able to give targeted anti-inflammatory medications for the specific inflammatory pathway, which is causing disease.

## Declaration of interests

The authors declare no competing interests.
